# Effect of Paediatric Rehabilitation in Infantile Systemic Hyalinosis: A Case Report

**DOI:** 10.7759/cureus.61866

**Published:** 2024-06-07

**Authors:** Nikita H Seth, H V Sharath, Raghumahanti Raghuveer, Moh'd Irshad Qureshi

**Affiliations:** 1 Neurophysiotherapy, Ravi Nair Physiotherapy College, Datta Meghe Institute of Higher Education and Research, Wardha, IND; 2 Paediatric Physiotherapy, Ravi Nair Physiotherapy College, Datta Meghe Institute of Higher Education and Research, Wardha, IND

**Keywords:** physiotherapy, respiratory complications, contracture, gene mutation, infantile systemic hyalinosis

## Abstract

Infantile systemic hyalinosis (ISH) is a very rare autosomal recessive disorder, which is characterized by a systemic build-up of hyaline material that causes extensive tissue destruction and functional impairment. The signs of this debilitating illness, which can involve organs, skin anomalies, and joint contractures, frequently appear in infancy. The paucity of available research on ISH emphasizes the need for all-encompassing management approaches to address the wide range of symptoms and enhance the overall quality of life for impacted babies. The interdisciplinary approach to ISH highlights the need for physiotherapy as a crucial element, with an emphasis on addressing the motor and developmental problems linked to the illness. Improving mobility and functional independence in newborns with ISH is facilitated by therapeutic exercises designed specifically for their needs. Here, we present a case of a six-month-old male child who visited a tertiary care center with complaints of minimal movements of all four limbs since birth with the inability to hold the neck. On examination, it was found that there were low-set ears with popular rashes and contractures over distal joints. Electromyography (EMG) and nerve conduction velocity (NCV) were done, which had abnormal findings suggestive of myopathy. On skin biopsy, it was confirmed that the child was suffering from ISH. Thus, the patient was referred to a physiotherapist. After six weeks of physiotherapy sessions, it was found that early and consistent physiotherapy interventions have been linked to a decrease in joint stiffness-related pain and discomfort, improving the affected infants' general comfort. Furthermore, physiotherapy interventions have a crucial role in supporting adaptive methods to get around physical restrictions, making it easier for infants with ISH to reach developmental milestones that could otherwise be difficult. Although there is little research on the effects of physical therapy on infants with ISH, new data indicate that a proactive, tailored physical therapy program can greatly enhance the functional ability of impacted children, improve their overall quality of life, and avert further problems. It is crucial to incorporate physiotherapy into the comprehensive care of infants diagnosed with ISH. This highlights the significance of timely diagnosis, interdisciplinary cooperation, and continuous research aimed at improving and optimizing physiotherapeutic therapies for this uncommon and crippling genetic illness.

## Introduction

Infantile systemic hyalinosis (ISH), an uncommon genetic illness, is characterized by aberrant hyaline material deposits throughout the body's tissues and organs. The disorder is autosomal recessive. Mutations in the ANTXR2 gene are the primary cause of this illness, which mainly affects babies. Many symptoms, such as joint contractures, skin thickening, painful nodules, gastrointestinal issues, and failure to flourish, are brought on by the aberrant hyaline deposits. Unfortunately, because ISH affects so many organs, it frequently results in death in young children [1]. Only a limited number of instances have been reported globally, indicating an exceedingly low frequency of ISH. Since it is an uncommon genetic illness, pinpointing the precise prevalence rate is difficult. Its rarity does, however, highlight how crucial early diagnosis and genetic counseling are for impacted families. Investigations on possible therapies and approaches to care for this debilitating illness are still in progress [2].

Subcutaneous lumps, skin anomalies, and joint contractures are the typical early signs of ISH. Joint stiffness that becomes worse over time limits movement, and skin abnormalities like thickness and hyperpigmentation occur. It is possible for subcutaneous nodules to ulcerate and get infected again. Affected internal organs, especially the gastrointestinal tract, may cause malabsorption and an inability to flourish. Complications from the heart could also occur. Regrettably, because of severe multi-organ failure, ISH frequently results in early mortality. Making a diagnosis is difficult and is usually verified by genetic testing [3].

A comprehensive clinical evaluation is necessary to diagnose ISH, which includes evaluating symptoms, such as joint contractures, skin lesions, and development failure. It is possible that lab tests will show more inflammatory markers. A skin biopsy is essential because it shows the distinctive hyaline deposits. The diagnosis is verified by genetic testing for ANTXR2 gene mutations. Examining joint involvement is made easier by imaging procedures like magnetic resonance imaging (MRI) and X-rays. Collaboration with pediatric specialists and geneticists is essential for a thorough examination of ISH due to its rarity. In order to effectively manage and provide supportive care for the multisystemic manifestations of this severe genetic illness, an early and accurate diagnosis is essential. Although there is presently no cure, management focuses on supportive care to reduce symptoms. In light of its rarity, promoting genetic counseling and increasing public awareness are essential components in treating this debilitating illness [4].

The management of ISH, a rare and severe genetic condition characterized by a systemic deposit of hyaline material, is heavily reliant on physiotherapy rehabilitation. Physiotherapy is intended to improve overall functionality by addressing motor and developmental problems in newborns with ISH. Exercises to increase joint flexibility, coordination, and muscle strength are examples of therapeutic therapies. Moreover, physiotherapists concentrate on encouraging both gross and fine motor abilities, supporting ideal posture, and treating discomfort brought on by tight joints. Despite the difficulties this crippling illness presents, early physiotherapy intervention helps newborns with ISH maximize their physical potential, promote independence, and enhance their quality of life [5].

## Case presentation

**Figure 1:**
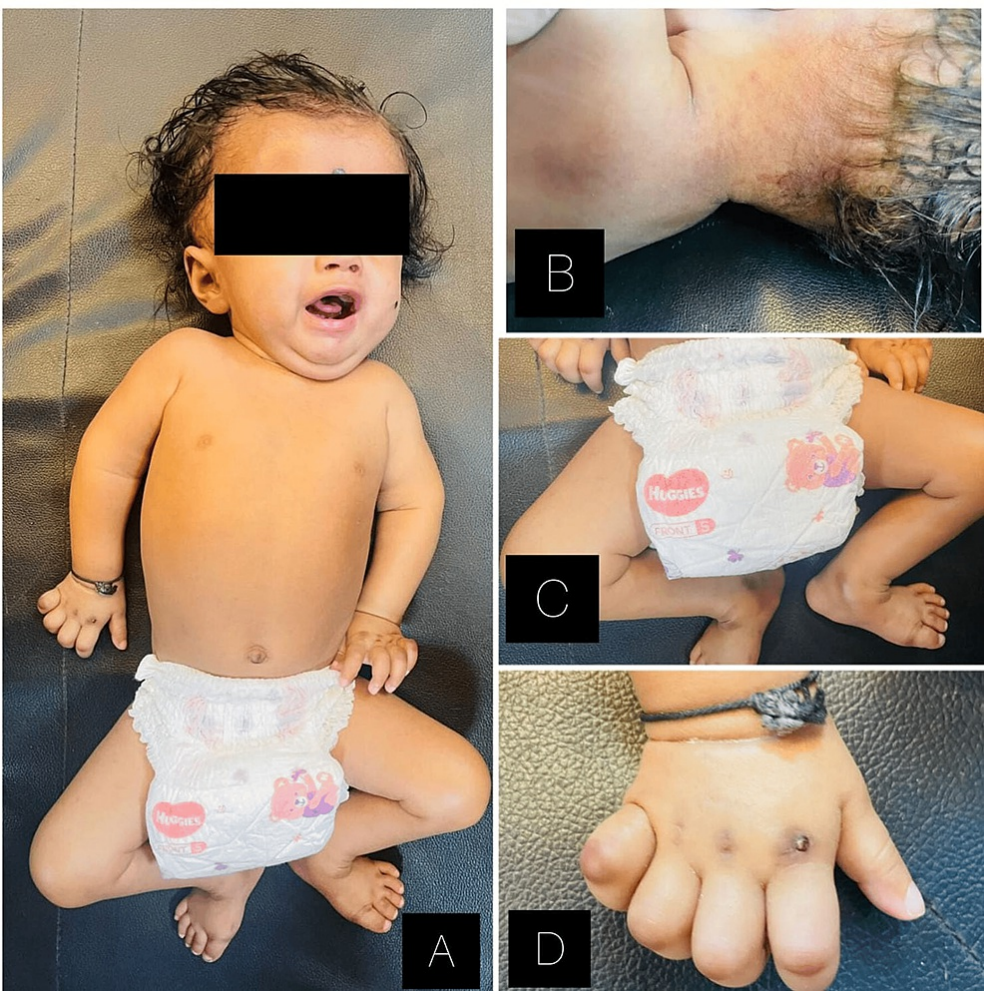
Clinical presentation of the child with infantile systemic hyalinosis A: Macrocephaly with low-set ears, B: multiple pustular lesions over the neck, C: hips fixed in abduction, D: contracture of distal joints.

A six-month-old male child came to the tertiary care hospital with complaints of minimal movements of all four limbs since birth. As narrated by the mother, she noticed that the baby had restricted movement of all four limbs at six weeks of life, which was more restricted in right than left. The baby was born by normal vaginal delivery with birth weight of 2.7 kg and cried after stimulation. The baby is born to parents with third-degree consanguineous marriage. Prenatal history revealed that the mother had oligohydroaminosis with intrauterine growth retardation. The immunization is completed as per age. On observation, the patient had low-set ears and macrocephaly with hips fixed in abduction with contractures in the distal joint with multiple pustular lesions over the neck, hyperpigmented nodule-like skin lesions over the dorsum of the fingers with sclerotic skin changes. On examination, the baby was vitally stable. The head circumference was 49 cm, suggestive of macrocephaly, and the length was 61 cm. On tone assessment, it was found that the upper limb has increased tightness with restricted joint integrity, and lower limbs could not be assessed since they were fixed in hip abduction, external rotation, and knee flexion. Reflexes were diminished with extensor plantar response, the primitive reflexes were sucking, rooting was present plantar, and palmar grasp was present. Moro’s reflex flexor withdrawal and cross extension were absent. The gross motor and fine motor milestones are delayed. Figure 1 depicts the clinical presentation of the child with ISH.

Investigations

A nerve conduction velocity study showed that compound motor action potential (CMAP) recorded from the bilateral tibial and median nerve showed lower CMAP with normal motor conduction velocity. Minimal F latency was not elicitable in the bilateral tibial and median nerve. An electromyography study was done on the left tibialis anterior and left quadriceps muscle. The spontaneous activity of all the muscles tested was electrically silent at rest, and voluntary activity showed reduced motor unit potential with early and complete recruitment seen in the muscle tested. Thus, electromyography (EMG) and nerve conduction velocity (NCV) studies were abnormal and suggestive of myopathy. Magnetic resonance imaging (MRI) of the brain revealed benign enlargement of subarachnoid spaces with mild cerebral atrophy. Skin biopsy showed normal epidermis and dermis with deposits of eosinophilic in the extracellular and perivascular space along with an increased amount of amorphous hyaline matrix suggestive of infantile systemic hyalinosis.

Intervention

The physiotherapy sessions were carried out for six weeks, five days per week. The rehabilitation protocol is given in Table 1.

**Table 1:** Physiotherapy management protocol

Sr. No.	Goal	Intervention	Rationale
1	To promote the range of motion	Gentle passive range of motion for all joints to prevent stiffness and contractures	Essential for maintaining joint integrity and preventing limitations in mobility
2	To encourage tummy time	Incorporate supervised tummy time sessions to enhance neck and upper body strength	Aids in developing postural control, strengthening muscles, and promoting motor skills
3	To stimulate visual tracking	Engage the child in activities with moving objects to encourage visual tracking	Enhances visual–motor coordination and supports the development of visual perception skills
4	To foster sensory integration	Introduce tactile stimuli through textured toys and surfaces to stimulate sensory development	Facilitates sensory processing, crucial for motor planning and overall cognitive development
5	To facilitate rolling movements	Gently guide the child in rolling activities to promote bilateral coordination and body awareness	Enhances spatial awareness, improves coordination, and supports the development of motor milestones
6	To support the development of grasping skills	To encourage reaching and grasping activities	Enhances fine motor skills, hand and eye coordination and promotes the development of pincer grasp essential for object manipulation
7	To facilitate social interaction	Structured play with caregivers to encourage social engagement and communication skill	Supports emotional development and social bonding and enhances overall quality of life
8	To educate caregivers on home exercises	Provide caregivers with a home exercise program and guidance on incorporating activities into daily routines	Empowers caregivers to actively participate in the child’s therapy and ensures consistent stimulation at home

**Figure 2:**
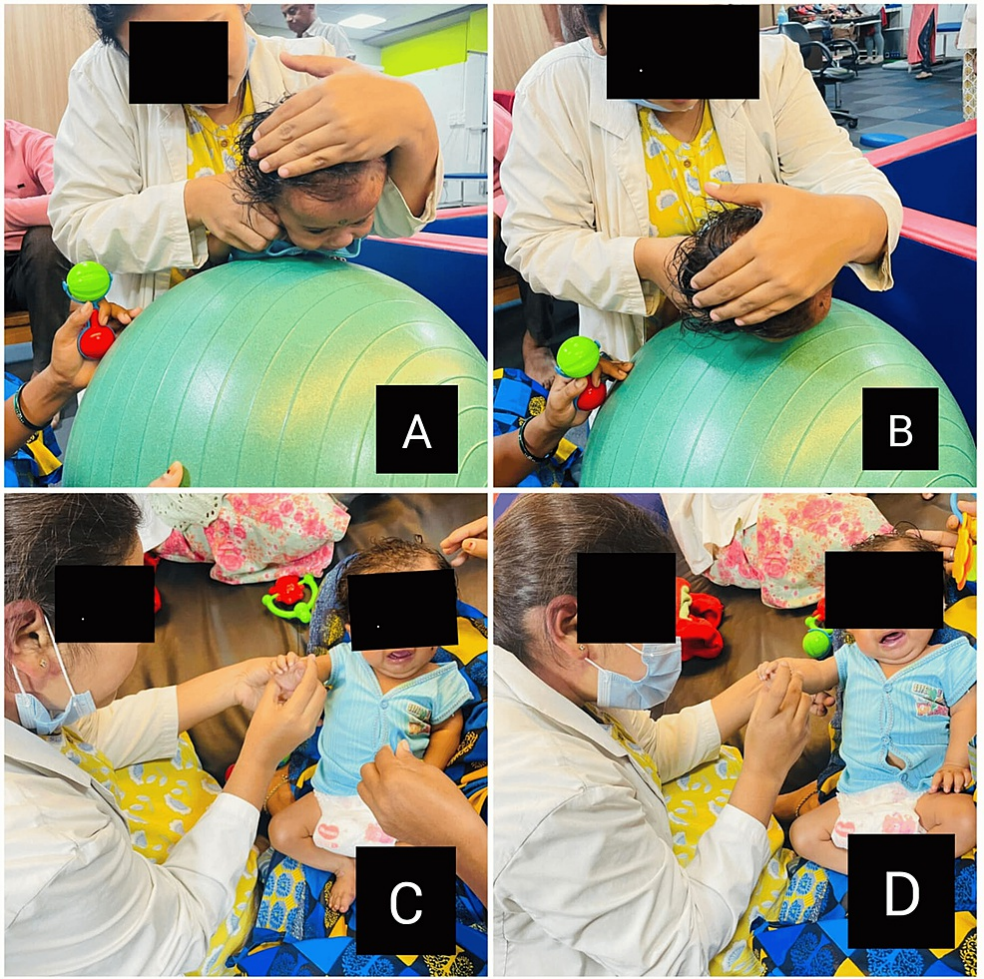
Demonstrating physiotherapy interventions in order to improve neck control and maintain joint integrity A and B: therapist demonstrating neck facilitation technique, C and D: gentle passive range of motion exercise to maintain joint integrity.

Figure 2 depicts the therapist performing physiotherapy interventions in order to improve neck control and maintain joint integrity.

Outcome measures

The outcome measures assessed were the Ballard Score, Hammersmith Infant Neurological Examination, and Infant Neurological International Battery (INFANIB). The timeline of assessment was baseline (pre-treatment), after six weeks (post-treatment), and the follow-up was taken after three weeks of termination of physiotherapy rehabilitation, as mentioned in Table 2.

**Table 2:** Outcome measures

Outcome measures scale	Pre-treatment	Post-treatment	Follow-up
Ballard score	17/50	22/50	24/50
Hammersmith Infant Neurological Examination	32/78	34/78	34/78
nfant Neurological International Battery (INFANIB)	23	26	26

## Discussion

An aberrant build-up of hyaline materials in different tissues causes a variety of crippling symptoms, which is the hallmark of the rare autosomal recessive illness known as ISH. Even though there is not a treatment for ISH, physiotherapy is extremely important for controlling symptoms and enhancing the lives of those who are impacted [6]. Numerous studies demonstrate how effective physiotherapy techniques are in treating the musculoskeletal symptoms associated with ISH. A study that was published in the Journal of Paediatric Rehabilitation Medicine showed how specialized physical therapy regimens, such as joint mobilization and stretching exercises, helped ISH patients' contractures and joint flexibility. The need for early and regular physiotherapeutic therapies to prevent progressive joint stiffness and abnormalities is highlighted by these findings [7].

In addition, physiotherapy is essential for helping newborns with ISH develop their motor skills. Improvements in gross and fine motor abilities were demonstrated in children who received early motor stimulation through physiotherapy, according to a study published in the *Journal of Child Neurology*. Physiotherapists can promote normal physical growth, minimize delays in developmental milestones, and improve motor function through well-planned workouts and activities [8].

Furthermore, pulmonary physiotherapy therapies are necessary since respiratory problems are common in individuals with ISH. Studies published in the *European Respiratory Journal* suggest that methods like breathing exercises and chest physiotherapy can help preserve lung health and guard against respiratory infections. In order to treat the multi-systemic character of ISH and enhance general respiratory health, this component of physiotherapy is essential.

## Conclusions

To sum up, physical therapy is a vital component of the all-encompassing approach to treating infantile systemic hyalinosis. Physiotherapy treatments have shown improvement in the quality of life for people with ISH by managing breathing difficulties, promoting motor development, and addressing musculoskeletal issues. Although there is not much data specifically on ISH, extrapolating from similar studies highlights how crucial it is to include physiotherapy in the all-encompassing care plan for those who have this uncommon and difficult condition.
